# Expansion of Clinical and Genetic Spectrum of *DDX3X* Neurodevelopmental Disorder in 23 Chinese Patients

**DOI:** 10.3389/fnmol.2022.793001

**Published:** 2022-03-22

**Authors:** Yuwei Dai, Zhuanyi Yang, Jialing Guo, Haoyu Li, Jiaoe Gong, Yuanyuan Xie, Bo Xiao, Hua Wang, Lili Long

**Affiliations:** ^1^Department of Neurology, Xiangya Hospital, Central South University, Changsha, China; ^2^National Clinical Research Center for Geriatric Disorders, Xiangya Hospital, Central South University, Changsha, China; ^3^Department of Neurosurgery, Xiangya Hospital, Central South University, Changsha, China; ^4^The Institute of Skull Base Surgery and Neurooncology at Hunan Province, Changsha, China; ^5^Department of Neurology, Hunan Children’s Hospital, Changsha, China; ^6^Hunan Provincial Maternal and Child Health Care Hospital, Changsha, China; ^7^NHC Key Laboratory of Birth Defects Research, Prevention and Treatment (Hunan Provincial Maternal and Child Health Care Hospital), Changsha, China

**Keywords:** *DDX3X*, intellectual disability, *DDX3X* syndrome, neuronal development, X-linked intellectual disability

## Abstract

**Aim:**

*De novo DDX3X* variants account for 1–3% of unexplained intellectual disability cases in females and very rarely in males. Yet, the clinical and genetic features of *DDX3X* neurodevelopmental disorder in the Chinese cohort have not been characterized.

**Method:**

A total of 23 Chinese patients (i.e., 22 female and 1 male) with 22 *de novo DDX3X* deleterious variants were detected among 2,317 probands with unexplained intellectual disability (ID) undertaking whole exome sequencing (WES). The age, sex, genetic data, feeding situation, growth, developmental conditions, and auxiliary examinations of the cohort were collected. The Chinese version of the Gesell Development Diagnosis Scale (GDDS-C) was used to evaluate neurodevelopment of *DDX3X* patients. The Social Communication Questionnaire (SCQ)-Lifetime version was applied as a primary screener to assess risk for autism spectrum disorder (ASD).

**Result:**

A total of 17 *DDX3X* variants were novel and 22 were *de novo*. Missense variants overall were only slightly more common than loss-of-function variants and were mainly located in two functional subdomains. The average age of this cohort was 2.67 (±1.42) years old. The overlapping phenotypic spectrum between this cohort and previously described reports includes intellectual disability (23/23, 100%) with varying degrees of severity, muscle tone abnormalities (17/23, 73.9%), feeding difficulties (13/23, 56.5%), ophthalmologic problems (11/23, 47.8%), and seizures (6/23, 26.1%). A total of 15 individuals had notable brain anatomical disruption (15/23, 65.2%), including lateral ventricle enlargement, corpus callosum abnormalities, and delayed myelination. Furthermore, 9 patients showed abnormal electroencephalogram results (9/23, 39.1%). Hypothyroidism was first noted as a novel clinical feature (6/23, 26.1%). The five primary neurodevelopmental domains of GDDS-C in 21 patients were impaired severely, and 13 individuals were above the “at-risk” threshold for ASD.

**Interpretation:**

Although a certain degree of phenotypic overlap with previously reported cohorts, our study described the phenotypic and variation spectrum of 23 additional individuals carrying *DDX3X* variants in the Chinese population, adding hypothyroidism as a novel finding. We confirmed the importance of *DDX3X* as a pathogenic gene in unexplained intellectual disability, supporting the necessity of the application of WES in patients with unexplained intellectual disability.

## Introduction

*DDX3X* (OMIM: 300160) locates in Xp11.4 and encodes a conserved ATP-independent DEAD-box RNA helicase, which is involved in transcription, splicing, RNA transport, and translation (Abdelhaleem, 2005; Garbelli et al., 2011). The DDX3X is composed of 622 amino acid residues containing two functional domains, namely, a helicase ATP-binding domain and a helicase C-terminal domain (Snijders Blok et al., 2015). *De novo DDX3X* variants account for 1–3% of unexplained intellectual disability (ID) or developmental delay (DD) (Deciphering Developmental Disorders Study., 2017; Maulik et al., 2011) and also perform as a highly plausible pathogenic gene for childhood apraxia of speech (CAS) (Hildebrand et al., 2020). Most cases of *DDX3X* variants have been reported in females but very rarely in males, and three previous large cohort studies have described heterogeneous clinical manifestations of *DDX3X* neurodevelopmental disorder, including ID or DD, dystonia, movement disorders, microcephaly, behavioral issues, feeding difficulties in infancy, and seizure (Snijders Blok et al., 2015; Wang et al., 2018; Johnson-Kerner et al., 2020; Lennox et al., 2020). However, the clinical and genetic features of *DDX3X* neurodevelopmental disorder in the Chinese cohort have not been described yet.

In this study, we elaborated on clinical manifestations of pathogenic variants of *DDX3X* in 23 patients (i.e., 22 female and 1 male) in the Chinese cohort and explore the association between genotypes and phenotypes.

## Materials and Methods

### Patients

With the support of the National Key Research and Development Program regarding the birth defect and developmental disorders screening (No. 2019YFC1005100), we collected whole exome sequencing (WES) data on 2,317 patients (1,622 males, 695 females, 5.33 ± 2.10 years old) with unexplained ID or DD and identified 23 *DDX3X* heterozygous variants in 23 patients by viewing those initial reports of WES. These patients further visited the Xiangya Hospital, Central South University, Hunan Provincial Maternal and Child Health Care Hospital, and Hunan Children’s Hospital between March 2018 and December 2020. Basic demographic information and detailed clinical data, including perinatal conditions, gender, date at birth, family history, genetic data, feeding situation, growth, and developmental conditions, were recorded clearly. Electroencephalography (EEG) and brain MRI were re-reviewed and reanalyzed by two experienced neurological physicians, and they were blind to the genetic results.

### Assessment

The Chinese version of the Gesell Development Diagnosis Scale (GDDS-C) was applied to assess the neurodevelopment of infants aged 0–6 years, and each participant calculated separate developmental quotient (DQ) of the five sub-domains, namely, adaptability, gross motor, fine motor, language, and social-emotional response. Based on the full-scale DQ results, the development of infants was classified as follows: normal (DQ ≥ 85), deficient (DQ < 75), and borderline (≥75 ∼ < 85). DQ in any single domain below 75 was considered deficient in this field (You et al., 2019). The GDDS-C was conducted by medical professionals in child health clinics (Yang, 2016).

The Social Communication Questionnaire (SCQ) Lifetime version was a brief, 40-item, parent-report clinical tool, which had been widely used as a primary screener to assess risk for autism spectrum disorder (ASD). It was based on a semi-structured parent interview conducted by a trained clinician or researcher. Each item in SCQ required a dichotomous “yes”/“no” response, and each item received a value of 1 point for abnormal behavior. Complete developmental history was needed to be the reference. The caregivers would indicate whether behaviors of Questions 2–19 had ever been presented and whether behaviors of Questions 20–40 were presented at the age 4 or evaluated these behaviors in the past half a year if the child was aged less than 4. Scores above the cutoff of 12 suggested individuals were above the “at-risk” threshold for ASD, and further extended evaluations should be undertaken (Marvin et al., 2017).

Differences between average scores on 2 scales of this cohort and respective cutoff value were statistically evaluated using a one-sample *t*-test, *p*-values less than 0.05 (*p < 0.05, ^**^p < 0.01, and ^***^p < 0.001) were considered significant.

### Genetic Analysis

We reanalyzed trio- or single WES data of all probands and their biological parents (19 for trio-WES). Sanger sequencing was conducted to validate whether the variant was *de novo*. The DDX3X transcript was referenced (NM_001193416.2, GRCh37/hg19). Sequenced reads were aligned to GRCh37/hg19 using the Burrows-Wheeler Aligner (BWA) (v.0.7.12) with default parameters. SAMtools (v0.1.12) was used to call the variants and the RefSeq Genomes database. The Genome Analysis Tool Kit (GATK 3.5) was used for local realignment and base quality score recalibration. Synonymous changes and single-nucleotide polymorphisms with a minor allele frequency greater than 5% were removed.^1^ Variant pathogenicity was interpreted based on the American College of Medical Genetics (ACMG) guidelines published in 2015 (Richards et al., 2015). The Genome Aggregation Database (GnomAD) was used to annotate the variants. Pathogenicity of the identified variants was predicted using several *in silico* predictors, including Polymorphism Phenotyping version 2 (Polyphen-2),^2^ Protein Variation Effect Analyzer (PROVEAN),^3^ Combined Annotation Dependent Depletion (CADD),^4^ and Sorting Intolerant From Tolerant (SIFT).^5^ Silico analysis data for missense DDX3X variants was shown in Supplementary material. Screening of neonatal genetic and metabolic diseases as a routine procedure was performed on all probands when they were born.

### Ethical Issues

This research was approved by the Ethics Committee of XiangYa Hospital, Central South University (Location: Hunan Province, P.R. China, Approval No.: 2019030496). Written consents for inclusion in this study and rights to use portraits of each proband were obtained from parents of all participants.

## Results

### Genomic Analysis

**FIGURE 1:**
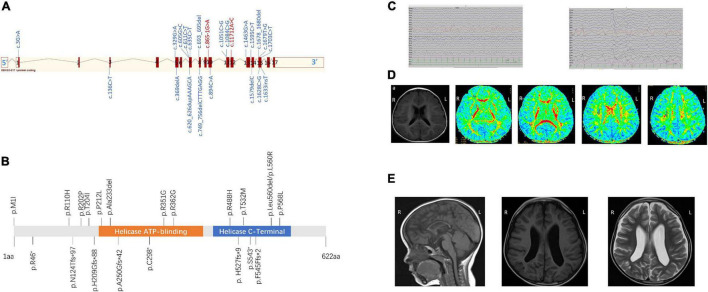
**(A)** Schematic view of the *DDX3X* exon structure based on NM_001193416. Red blocks represent exons, and the exon number is listed on each exon. cDNA change is listed for each variant. Splice site mutations are shown in red font. **(B)** Location of amino acid substitutions in DDX3X (NM_ 001193416.2). Missense and in-frame deletions (top, 13), frameshift, and non-sense variants (bottom, 8). DDX3X contains two subdomains, a helicase ATP-binding domain (orange bar) and a helicase C-terminal domain (blue bar). **(C)** The latest electroencephalogram (EEG) and of Female 17. Abnormal EEG presentation, multiple slow waves in bilateral occipital lobes. **(D)** Brain magnetic resonance imaging (MRI) of Female 19 at the age of 1 year and 1 month **(A–E)** Axial position T1-weighted images show normal sulci and gyri, Axial position diffusion tensor imaging (DTI) images show delayed of white matter of frontal lobe and centrum semiovale myelination. Numbers 1–8 represent genu of the corpus callosum, white matter of the frontal lobe, anterior limb of the internal capsule, posterior limb of the internal capsule, splenium of the corpus callosum, occipital lobe, and centrum semiovale (7 and 8), respectively. **(E)** Sagittal image shows diffuse thinning of the corpus callosum of Female 13 at the age of 1 year and 3 months **(A)**. MRI **(B)** T1 and **(C)** T2 axial slices showed widened bilateral lateral ventricles of Female 6 at the age of 3 years and 10 months.

Among 2,317 individuals studied by WES who had unexplained ID, 23 deleterious variants in *DDX3X* were detected, 22 females were found to carry *de novo* variants in *DDX3X*, and 1 male was identified to carry an inherited variant in *DDX3X* from his asymptomatic mother. Furthermore, 17 were novel variants, and the remaining 6 variants were reported previously [c.1595C > T (Lennox et al., 2020); c.136C > T (Snijders Blok et al., 2015); c.865-1G > A (Wang et al., 2018); c.1703C > T (Snijders Blok et al., 2015); c.1463G > A (Lennox et al., 2020); c.1678_1680del (Snijders Blok et al., 2015)]. Of the 23 identified variants in *DDX3X*, 11 were missense variants, 2 were in-frame deletions, 2 were splice site variants, 5 were frameshift variants, and 3 were nonsense variants (Figure 1A). According to the guidelines set out by the ACMG, 22 variants were interpreted as pathogenic or likely pathogenic variants, and 1 variant was of uncertain significance (VUS) (Table 1).

**TABLE 1:** Clinical interpretation of variants detected in *DDX3X* by the ACMG guideline.

Patient	Genotype	Inheritance	Variant (NM_001193416)	Evidence of pathogenicity based on ACMG guideline				Category
				Very strong	Strong	Moderate	Supporting	
Female 1	Het	*De novo*	c.1084C > G p.(R362G)	/	PS2	PM1 + PM2 + PM5	PP3	Pathogenic
Female 2	Het	*De novo*	c.635C > T; p.(P212L)	/	PS2	PM1 + PM2 + PM5	PP3	Pathogenic
Female 3	Het	*De novo*	c.1579delC p.(H527fs*9)	PVS1	PS2	PM2	/	Pathogenic
Female 4	Het	*De novo*	c.1171-2A > C; ?	PVS1	PS2	PM2	/	Pathogenic
Female 5	Het	*De novo*	c.369delA; p.(N124Tfs*97)	PVS1	PS2	PM2	/	Pathogenic
Female 6	Het	*De novo*	c.1051C > G; p.(R351G)	/	PS2	PM1 + PM2	PP3	Likely pathogenic
Female 7	Het	*De novo*	c.611C > T; p.(T204I)	/	PS2	PM2	PP3	Likely pathogenic
Female 8	Het	*De novo*	c.1595C > T; p.(T532M)	/	PS1 + PS2	PM1 + PM2	PP3	Pathogenic
Female 9	Het	*De novo*	c.749_756del CTTTGAGG; p.(A250Gfs*42)	PVS1	PS2	PM2	/	Pathogenic
Female 10	Het	*De novo*	c.136C > T; p.(R46*′)	PVS1	PS2	PM2	/	Pathogenic
Female 11	Het	*De novo*	c.865-1G > A; ?	PVS1	PS2	PM2		Pathogenic
Female 12	Het	*De novo*	c.693_695del p.(Ala233del)	/	PS2	PM2 + PM4	PP3	Likely pathogenic
Female 13	Het	*De novo*	c.894C > A; p.(C298*)	PVS1	PS2	PM2	/	Pathogenic
Female 14	Het	*De novo*	c.1633insT; p.(F545Ffs*2)	PVS1	PS2	PM2	/	Pathogenic
Female 15	Het	*De novo*	c.1678_1680del; p.(L560del)	/	PS2	PM1 + PM2 + PM4	PP3	Pathogenic
Female 16	Het	*De novo*	c.1703C > T; p.(P568L)	/	PS1 + PS2	PM2	PP3	Pathogenic
Female 17	Het	*De novo*	c.1679T > G; p.(L560R)	/	PS2	PM1 + PM2	PP3	Likely pathogenic
Female 18	Het	*De novo*	c.1463G > A; p.(R488H)	/	PS1 + PS2	PM1 + PM2	PP3	Pathogenic
Female 19	Het	*De novo*	c.620_626 dupAAAGCA; p.(His209Glnfs*88)	PVS1	PS2	PM2	/	Pathogenic
Female 20	Het	*De novo*	c.3G > A; p.(M1I)	PVS1	PS2	PM2	/	Pathogenic
Female 21	Het	*De novo*	c.1628C > G; p.(S543*)	PVS1	PS2	PM2	/	Pathogenic
Female 22	Het	*De novo*	c.605G > C p.(R202P)	/	PS2	PM2	PP3	Likely pathogenic
Male 1	Het	Inherited from his mother	c.329G > A; p.(R110H)	/	/	PM2	PP3	Variant of Uncertain Significance

*ACMG, American College of Medical Genetics; PVS, pathogenic very strong; PS, pathogenic strong; PM, pathogenic moderate; PP, pathogenic supporting.*

All 23 identified variants were likely to cause changes in the DDX3X protein, 6 of which were in the helicase ATP-binding domain (i.e., p.Ala233del, p.P212L, p.R351G, p.R362G, p.A250Gfs*42, and p.C298* while 8 were in the helicase C-terminal domain (i.e., p.R488H, p.T532M, p.Leu560del, p.L560R, p.P568L, p. H527fs*9, p.S543*, and p.F545Ffs*2) (Figure 1B).

### Clinical Features

Table 2 shows the clinical features of 23 participants. The average age of this cohort was 2.67 (±1.42) years old. In the cohort of 23 patients with *DDX3X* variants, all of them meet the criteria for ID or DD (23/23, 100%), ranging from mild to severe. Muscle tone abnormalities (17/23, 73.9%), including isolated hypotonia, hypertonia, or mixture of hypertonia and hypotonia, microcephaly (9/23, 39.1%), feeding difficulties, or low weight gain (13/23, 56.5%), associated with ophthalmologic problems (11/23, 47.8%), were the most typical clinical characteristics. Movement disorders (9/23, 39.1%), seizures (6/23, 26.1%), behavior issues (5/23, 21.7%), cardiac abnormalities (3/23, 13.0%), and hearing impairment (2/23, 8.7%) were observed in this cohort. Furthermore, six patients presented with hypothyroidism (6/23, 26.1%).

**TABLE 2:** Summary of demographic information and phenotypic features in 23 patients with *DDX3X* variants.

Patient	Female 1	Female 2	Female 3	Female 4	Female 5	Female 6	Female 7	Female 8	Female 9	Female 10	Female 11
Current age (years, months)	2y3m	1y7m	1y9m	1y	4y	4y3m	1y	3y5m	3y6m	3y	2y4m
Perinatal conditions	Normal	Normal	Normal	Normal	MLBW	Normal	Normal	Normal	Normal	Normal	Normal
ID/DD	+	+	+	+	+	+	+	+	+	+	+
Weight	−SD	−3SD	−3SD	−2SD	Normal	Normal	−SD	−2SD	−2SD	Normal	−3SD
Height	−2SD	Normal	Normal	Normal	+ SD	Normal	Normal	−SD	Normal	+ SD	−SD
Speech	Single words	Minimally verbal	Single words	Minimally verbal	Single words	Single words	Single words	Minimally verbal	Single words	Single words	Minimally verbal
Age at walking	2y2m (with rollator)	No	No	No	3	1y5m (wide base gait)	No	2y9m (wide base gait)	1y8m	No	No
Tone abnormalities	Hypertonia	Normal	Hypotonia	Mixture	Hypotonia	Mixture	Hypotonia	Hypotonia	Normal	Normal	Hypertonia
Movement disorders	+ ataxia	No	No	No	No	+ abnormal gait	No	+ abnormal gait	+ dystonia	No	+ dyskinesia
Seizures	Absence seizures	No	No	No	No	No	No	No	Atonic seizures	No	Absence seizure
Microcephaly	No	No	No	No	No	No	No	No	+, −2SD	+, −3SD	+, −2SD
Ophthalmologic problems	Refractive errors	No	No	No	Amblyopia	Refractive errors	No	No	Refractive errors	Refractive errors	Refractive errors
Behavior issues	No	No	No	No	ASD	No	Hyperactivity	No	No	No	ASD
SCQ Lifetime	20	NA	NA	13	24	13	NA	22	17	10	21
Others	PFO	Feeding difficulties	Constipation	Constipation, Inability to chew; Hearing impairment	No	No	Feeding difficulties	Feeding difficulties, constipation	Feeding difficulties, constipation	No	Feeding difficulties; constipation
Endocrine abnormalities (TSH level)	Hypothyroidism (8.97 mIU/L)	No (Normal)	No (Normal)	Hypothyroidism (16.53 mIU/L)	No	No	No	No	No	No	Hypothyroidism (9.21 mIU/L)
MRI findings	Ventricular enlargement	Ventricular enlargement	Ventricular enlargement	Normal	Normal	Ventricular enlargement	Ventricular enlargement	White matter volume reduction; subdural effusion	Ventricular enlargement	Normal	Normal
Current age (years, months)	1y4m	6y10m	1y4m	2y	5y	2y10m	3y	3y	1y8m	1y10m	1y10m	1y4m
perinatal conditions	Normal	Normal	Normal	MLBW	MLBW	Normal	Normal	Neonatal jaundice	Normal	Normal	Normal	Neonatal jaundice
ID/DD	+	+	+	+	+	+	+	+	+	+	+	+
Weight	−SD	Normal	−SD	−3SD	−2SD	−3SD	−SD	−SD	−2SD	Normal	Normal	−2SD
Height	Normal	Normal	−SD	Normal	−2SD	−2SD	Normal	Normal	Normal	+ 2SD	−SD	−SD
Speech	Single words	Single words	Single words	Minimally verbal	Minimally verbal	Minimally verbal	Minimally verbal	Single words	Minimally verbal	Single words	Minimally verbal	Single words
Age at walking	No	No	No	No	No	No	No	No	No	1y8m	No	No
Tone abnormalities	Hypertonia	Hypotonia	Normal	Hypotonia	Mixture	Hypotonia	Hypotonia	Normal	Hypotonia	Normal	Hypotonia	Hypotonia
Movement disorders	+ dyskinesia	No	+ ataxia	No	+ dystonia	No	No	No	No	+ dystonia	No	No
Seizures	Absence seizures	No	No	No	Focal partial seizure	Infantile spasms	No	Absence seizures	No	No	No	No
Microcephaly	No	No	+, −2SD	+, −2SD	+, −2SD	+, −2SD	No	+,−2SD	+, −2SD	No	No	No
Ophthalmologic problems	No	No	Nystagmus	No	No	Strabismus	Refractive errors	No	No	No	Amblyopia	Refractive errors
Behavior issues	No	No	No	No	No	No	No	No	Hyperactivity	No	No	ASD
SCQ lifetime	NA	11	NA	13	17	20	18	14	NA	10	12	19
Others	No	Constipation	Constipation	PFO; hearing impairment; feeding difficulties, constipation	Inability to chew	Inability to chew	Normal	Feeding difficulties, constipation	Feeding difficulties, constipation	Feeding difficulties, constipation	Atrial septal defect; inability to chew	Feeding difficulties; constipation
Endocrine abnormalities (TSH level)	No	No	No	Hypothyroidism (7.74 mIU/L)	Hypothyroidism (5.8 mIU/L)	No	No	Hypothyroidism (8.35 mIU/L)	No	No	No	No
MRI findings	Delayed myelination	Corpus callosum abnormalities	Normal	Ventricular enlargement	Corpus callosum abnormalities	Ventricular enlargement	Ventricular enlargement	Delayed myelination	Normal	Normal	Normal	Ventricular enlargement

*ID, intellectual disability; DD, developmental disability; MLBW, mature low birth weight; Mixture, Mixed hypo and hypertonia; PFO, patent foramen ovale; ASD, atrial septal defect; SD, standard deviation; +, positive; NO, negative; NA, not available; TSH, thyroid-stimulating hormone.*

#### Intellectual Disability or Developmental Delay

Only 2 patients (i.e., Female 14 and Female 18) could raise their heads at the age of 3 months, and 20 patients (except Female 7, Female 10, and Female 21) could not walk independently before the age of 2 years, all of them had poor motor coordination. All parents complained their children showed poor performance in language or speech function. Nearly 50% could express their simple needs in no more than four words and only say several single words. The two eldest individuals above 5 years (i.e., Female 13 and Female 16) had not developed speech ability but could just follow simple instructions.

#### Seizures and Electroencephalography Monitoring

All of them underwent scalp EEG monitoring, and 9 of them showed abnormal profiles. Slow background activity was observed in 7 out of 23 patients. Focal epileptiform discharges were detected in two patients, and generalized spike waves and sharp waves were detected in four patients. Hyperarrhythmia, associated with multifocal epileptiform discharges, was prevalent in one patient. In 6 individuals with seizures (i.e., Female 1, Female 9, Female 11, Female 16, Female 17, and Female 19), their age at the onset of seizures ranged from 5 to 14 months. Atonic seizures occurred in 1, absence seizures in 3, focal partial seizures in 1, and infantile spasms in 1. Female 17 was diagnosed with infantile spasms induced by fever at the age of 5 months and recurred at the age of 13 months. They had a good response to antiepileptic drugs and no seizures in 6 months. Figure 1C shows the latest EEG of Female 17.

#### Feeding Difficulties

Among the 13 individuals with feeding difficulties or low weight in our cohort, 8 of their parents reflected that they were intolerant of lactose and allergic to multiple high-protein food sources. Constipation and chewing weakness were common manifestations in 10 cases. Furthermore, 7 of 13 individuals were vitamin B-deficient, but none of them was hyperhomocysteinemia.

#### Endocrine Abnormalities

Thorough endocrine hormone examinations were conducted in 23 patients because of poor growth and neurocognitive development. None of them showed abnormal sex hormone levels. Furthermore, six patients had lower levels of thyroid hormones (THs), and thyroid ultrasound showed normally located thyroid glands. They all received TH supplementation.

#### Magnetic Resonance Imaging Findings

All patients completed MRI at different ages. A total of 15 individuals had notable brain anatomical disruption in which, 10/15 had a lateral ventricle enlargement, 2/15 had corpus callosum abnormalities, 2/15 had delayed myelination, and 1/15 had white-matter volume reduction. Figure 1D shows brain MRI of Female 19, and (Figure 1E) shows brain MRI of Female 6 and Female 13.

### Assessments

**FIGURE 2:**
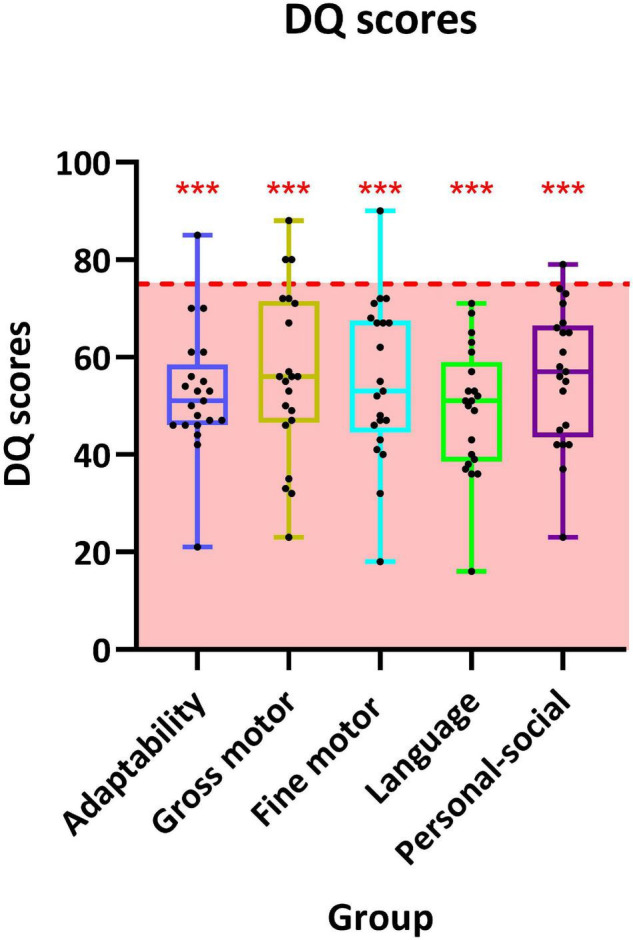
Distribution of developmental quotient (DQ) scores of 19 individuals in the five sub-domains of the Chinese version of Gesell Development Diagnosis Scale (GDDS-C). The interquartile ranges are shown as a box-and-whisker plot. For one-sample *t*-test, the critical value is 75 (*** represents *p* < 0.001).

A total of 21 patients have undergone GDDS-C and were counted DQ in five separate domains. The highest scored domain was “gross motor” (56.1 ± 17.2), and the lowest scored domain was “language” (49.1 ± 13.2). The remaining scored domains were “fine motor” (56.1 ± 17.2), “personal-social” (56.0 ± 14.2), and “adaptability” (52.7 ± 12.7) successively. Scores in all 5 domains were significantly below the critical value (75), in which *p*-value was < 0.01. DQ of each participant is listed in Figure 2.

A total of 17 patients completed the SCQ-Lifetime version, and 13 individuals who were above the cutoff value (12) were at the risk of ASD, a comprehensive evaluation for ASD was warranted. Significant deviation was found in the mean score in the SCQ-Lifetime version of 17 participants (16.1 ± 4.4, *p* = 0.002) compared with the cutoff value.

## Discussion

In this report, we described a Chinese cohort of patients with *DDX3X* variants (*n* = 23, 22 confirmed *de novo*), and we are the first study to pinpoint clinical and genetic characteristics in the Chinese population. Among the 23 *DDX3X* variants in our cohort, 17 were novel variants and 14 variants were located in two functional domains of DDX3X (9 were amino acid variants and 5 were truncating variants). Significant differences in sex composition of *DDX3X* neurodevelopmental disorder have been noted (22 female and 1 male). ID was considered as a universal feature of *DDX3X* neurodevelopmental disorder in our study, followed by tone abnormalities, microcephaly, feeding difficulties, and seizures. Major aspects of neural development were assessed and quantified using the GDDS-C, and mean scores of five domains were significantly lower than the critical value of 75 (all *p*-value < 0.05), and language domain was impaired strikingly. Hypothyroidism was reported in 6 patients with *DDX3X* variants for the first time. Altogether, our study expanded the clinical and genetic spectrum associated with *DDX3X* neurodevelopmental disorder and evaluated the degree of developmental delay by a standardized scale. It highlighted that WES was necessary for those unexplained ID individuals.

DDX3X, an RNA-binding protein of the DEAD-box family encoded by the *DDX3X* gene (Abdelhaleem, 2005), acts as a translational regulator (Lai et al., 2008; Lee et al., 2008), particularly for mRNAs with highly structured 5′ untranslated regions (UTRs) (Chen et al., 2018) and for repeat-associated non-AUG translation (Cheng et al., 2019; Linsalata et al., 2019). DDX3X is also the key component of ribonucleoprotein (RNP) granules composed of mRNA and protein (Huang et al., 2019), a pathological hallmark of many neurodegenerative diseases (Ramaswami et al., 2013). Dd3x plays an indispensable role in mouse embryogenesis, synaptogenesis, and brain development (Chen et al., 2016; Lennox et al., 2020). Dd3x neural stem cells knockout at embryonic day (E) 9.5 in mice hampered brain growth, accompanied by seizures and ataxia (Patmore et al., 2020). Boitnott et al. (2021) generated a Ddx3x haploinsufficient mouse (Ddx3x^±^ female) with construct validity for *DDX3X* loss-of-function mutations. The Ddx3x^±^ mice showed global development delay and evolved into behavioral anomalies in adulthood (Boitnott et al., 2021).

Comparing with three previously reported cohort studies of *DDX3X* neurodevelopmental disorder, we found a certain degree of phenotypic overlap but some special points (Snijders Blok et al., 2015; Wang et al., 2018; Lennox et al., 2020; Table 3). ID was still a general phenotypic feature of patients with *DDX3X* variants in both our study and previous reports. Similarly, compared with healthy controls, the mean score in GDDS-C of the cohort could reflect global DD. The worst performance in the language subdomain of GDDS-C consolidated language impairments as the most prominent clinical feature of *DDX3X* neurodevelopmental disorder (Johnson-Kerner et al., 2020). Ophthalmologic problems including refractive errors, nystagmus, strabismus, and amblyopia were presented in 11/23 (47.8%) patients. Previous studies have shown that a variety of eye phenotypes including hypoplasia of the eye or absence of one or both eyes in functional studies of ddx3x knockdown in zebrafish, suggesting deleterious variants in *DDX3X*, may hamper eye function (Snijders Blok et al., 2015; Kellaris et al., 2018). The SCQ-Lifetime version scores indicated an increased risk for ASD in *DDX3X* neurodevelopmental disorder, suggesting the necessity of screening for behavior problems by trained behavioral pediatricians.

**TABLE 3:** Comparison of clinical characteristics in our cohort and three previously published cohorts.

Clinical findings	Numbers (percentage,%)[our study]	Numbers (percentage,%) (Snijders Blok et al., 2015)	Numbers (percentage,%) (Wang et al., 2018)	Numbers (percentage,%) (Lennox et al., 2020)
**Neurological**				
Intellectual disability (ID) or developmental delay (DD)	23/23 (100%)	38/38 (100%)	28/28 (100%)	106/106 (100%
Hypotonia	11/23 (47.8%)	29/38 (76%)	19/28 (68%)	54/93 (58%)
Hypertonia	3/23 (10.7%)	N/A	2/12 (17%)	5/93 (5%)
Mixed hypo and hypertonia	3/23 (10.7%)	N/A^a^	N/A	31/93 (33%)
Movement disorders	9/23 (39.1%)	17/38 (45%)	17/28 (61%)	18/83 (22%)
Seizures	6/23 (26.1%)	6/38 (16%)	N/A	17/93 (18%)
Ophthalmologic problems	11/23 (47.8%)	13/38 (34%)	9/28 (32%)	29/92 (31.5%)
Microcephaly	9/23 (39.1%)	12/38 (32%)	7/28 (25%)	34/90 (38%)
Behavior issues	5/23 (21.7%)	20/38 (53%)	6/28 (21%)	N/A
**Others**				
Cardiac abnormalities	3/23 (13.0%)	N/A	5/27 (71%)	13/90 (14%)
Feeding difficulties or low weight	13/23 (56.5%)	12/38 (32%)	N/A	N/A
Hearing impairment	2/23 (8.7%)	3/38 (3%)	N/A	4/78 (5%)
**Imaging findings**				
Corpus callosum abnormalities	2/23 (8.7%)	13/37 (35%)	18/20 (90%)^b^	77/89 (87%)
Ventricular enlargement	10/23 (43.5%)	13/37 (35%)		61/89 (68%)
Cortical malformation	3/23 (13.1%)	4/37 (11%)		50/89 (56%)
Delayed myelination	2/23 (8.7%)	N/A		N/A

*^a^In Snijders Blok et al. (2015), movement disorders include spasticity.*

*^b^In Wang et al. (2018), imaging findings refer to structural brain abnormalities.*

Furthermore, we noticed hypothyroidism in 6 patients. This novel or rare clinical feature was not previously reported in the original description of *DDX3X* neurodevelopmental disorder. Poor nutrient absorption and feeding difficulty could make them at increased risk of iodine deficiency, which could be an extrinsic factor causing hypothyroidism in these younger children (Bauer and Wassner, 2019). THs play an essential role in the growth and metabolic homeostasis in humans as well as in animals (Prezioso et al., 2018). Triiodothyronine (T3), the active form of thyroid hormone, acts on its nuclear receptor and modulates target gene transcription (Kumar et al., 2015). Even a 25% reduction in *DDX3X* levels strongly perturbs neurogenesis, suggesting the high dose-dependency of embryonic cortical development to DDX3X (Lennox et al., 2020). Defective RNA metabolism was considered as the potential mechanism through which *DDX3X* missense variants hamper fetal brain cortical development (Kumar et al., 2015; Lennox et al., 2020). We speculated that TH deficiency may intensify the adverse effect on RNA metabolism caused by *DDX3X* missense variants. Appropriate TH supplementation in *DDX3X* patients with hypothyroidism could be worth trying, but overtreatment or prophylactic hormonal therapy should be avoided because the higher dose of TH supplementation could worsen the outcome (Tuhan et al., 2016). Therefore, it is crucial to have a close follow-up in *DDX3X* patients with hypothyroidism. Hypothyroidism has not been verified in animal models with *in vivo* depletion of Ddx3x.

Many study reports have tried to establish the connection between the severity of clinical phenotypes and the location and type of variants and obtained two main findings (Lennox et al., 2020). First, the same recurrent *de novo* variants were more likely to have similar phenotypes. Recurrent amino acids changes, including R326, I415, and T532, all could cause polymicrogyria (PMG) (Abdelhaleem, 2005; Tuhan et al., 2016; Lennox et al., 2020). Besides the 11 individuals with PMG, 10 were missense variants and one was in-frame deletion, underscoring a striking association between missense variants and severe cerebral anatomical phenotypes, like PMG or dysgyria (Lennox et al., 2020).

Regrettably, no PMG or dysgyria was observed in our cohort, nor the previously reported *DDX3X* variants relating with PMG. However, we found that females with missense or in-frame deletion *DDX3X* variants (10/11, 90.9%) were more likely to have abnormal brain structural MRI compared with those with LOF variants (4/11, 36.3%). This could be interpreted by different pathogenic mechanisms. Many aberrant truncating mRNAs (i.e., frameshift or non-sense variants) might undergo non-sense-mediated RNA decay (NMD) and resulted in a haploinsufficiency effect, while a subset of missense variants could function in a dominant-negative manner (Chen et al., 2020; Lennox et al., 2020). Further investigations about gain-of-function mechanism behind certain missense mutations will be carried out by modeling missense mutations in mice. Multiple malignancies have a solid association with somatic *DDX3X* variants, like malignant melanoma and medulloblastoma (Phung et al., 2019; Patmore et al., 2020). Even though no malignancy was reported in our cohort yet, regular cancer screening is still quite necessary. Finally, a small sample size and a relatively small number of novel phenotypes, such as hypothyroidism, were the main limitations of this study.

In summary, we identified 23 unrelated Chinese patients with causal variants in *DDX3X* and expanded the knowledge of these increasingly recognized ID disorders. Our study delineated many clinical characteristics of the Chinese cohort with *DDX3X* variants, largely overlapping with phenotypic spectrum in previously reported studies, but hypothyroidism was first noted as a novel clinical feature. Overall, missense variants were only slightly more common than loss-of-function variants and were mainly located in two functional subdomains. The *DDX3X* missense variants may have a certain association with abnormal brain anatomical structures. Given the heterogeneous clinical manifestations and involvement of the nervous system and non-nervous systems, unexplained ID in both males and females should take the use of multigene panels that include *DDX3X* or WES into consideration.

## Data Availability Statement

Sequencing data involved in the study are available through the Genbank repository (https://www.ncbi.nlm.nih.gov/bioproject/PRJNA795095). There are restrictions to the full availability of sequencing data of the research participants due to privacy and ethical/legal issues. The data that support the findings of this study are available from the corresponding author, upon reasonable request.

## Ethics Statement

The studies involving human participants were reviewed and approved by the Ethics Committee of XiangYa Hospital, Central South University. Written informed consent to participate in this study was provided by the participants’ legal guardian/next of kin. Written informed consent was obtained from the individual(s), and minor(s)’ legal guardian/next of kin, for the publication of any potentially identifiable images or data included in this article.

## Author Contributions

YD, ZY, and LL: study design, analysis and revision of the manuscript. YD, ZY, and JGu: follow-up of patient’s information. HL, JGo, and YX: reanalysis of WES data and original draft preparation. YD, BX, HW, and LL: collection of clinical and WES data. All authors contributed to the article and approved the submitted version.

## Conflict of Interest

The authors declare that the research was conducted in the absence of any commercial or financial relationships that could be construed as a potential conflict of interest.

## Publisher’s Note

All claims expressed in this article are solely those of the authors and do not necessarily represent those of their affiliated organizations, or those of the publisher, the editors and the reviewers. Any product that may be evaluated in this article, or claim that may be made by its manufacturer, is not guaranteed or endorsed by the publisher.

## Footnotes

1
http://www.ncbi.nlm.nih.gov/projects/SNP

2
http://genetics.bwh.harvard.edu/pph2/

3
http://provean.jcvi.org

4
https://cadd.gs.washington.edu/

5
http://sift.jcvi.org

## References

[B1] Abdelhaleem M. (2005). RNA helicases: regulators of differentiation. *Clin. Biochem.* 38 499–503. 10.1016/j.clinbiochem.2005.01.010 15885226

[B2] Bauer A. J. Wassner A. J. (2019). Thyroid hormone therapy in congenital hypothyroidism and pediatric hypothyroidism. *Endocrine* 66 51–62. 10.1007/s12020-019-02024-6 31350727

[B3] Boitnott A. Garcia-Forn M. Ung D. C. Niblo K. Mendonca D. Park Y. (2021). Developmental and Behavioral Phenotypes in a Mouse Model of *DDX3X* Syndrome. *Biol. Psychiatr.* 90 742–755. 10.1016/j.biopsych.2021.05.027 PMC8571043 34344536

[B4] Chen H. H. Yu H. I. Tarn W. Y. (2016). DDX3 Modulates Neurite Development via Translationally Activating an RNA Regulon Involved in Rac1 Activation. *J. Neurosci.* 36 9792–9804. 10.1523/JNEUROSCI.4603-15.2016 27656019 PMC6705563

[B5] Chen H. H. Yu H. I. Yang M. H. Tarn W. Y. (2018). DDX3 Activates CBC-eIF3-Mediated Translation of uORF-Containing Oncogenic mRNAs to Promote Metastasis in HNSCC. *Cancer Res.* 78 4512–4523. 10.1158/0008-5472.CAN-18-0282 29921696

[B6] Chen Y. Liu K. Y. Yang Z. L. Li X. H. Xu R. Zhou H. (2020). A de novo *DDX3X* Variant Is Associated With Syndromic Intellectual Disability: case Report and Literature Review. *Front. Pediatr.* 8:303. 10.3389/fped.2020.00303 PMC7344189 32714884

[B7] Cheng W. Wang S. Zhang Z. Morgens D. W. Hayes L. R. Lee S. (2019). CRISPR-Cas9 Screens Identify the RNA Helicase *DDX3X* as a Repressor of C9ORF72 (GGGGCC)n Repeat-Associated Non-AUG Translation. *Neuron* 104 885.e–898.e. 10.1016/j.neuron.2019.09.003 31587919 PMC6895427

[B8] Deciphering Developmental Disorders Study. (2017). Prevalence and architecture of de novo mutations in developmental disorders. *Nature* 542 433–438. 10.1038/nature21062 28135719 PMC6016744

[B9] Garbelli A. Beermann S. Di Cicco G. Dietrich U. Maga G. (2011). A motif unique to the human DEAD-box protein DDX3 is important for nucleic acid binding, ATP hydrolysis, RNA/DNA unwinding and HIV-1 replication. *PLoS One* 6:e19810. 10.1371/journal.pone.0019810 PMC3093405 21589879

[B10] Hildebrand M. S. Jackson V. E. Scerri T. S. Van Reyk O. Coleman M. Braden R. O. (2020). Severe childhood speech disorder: gene discovery highlights transcriptional dysregulation. *Neurology* 94 e2148–e2167. 10.1212/WNL.0000000000009441 32345733

[B11] Huang M. Tailor J. Zhen Q. Gillmor A. H. Miller M. L. Weishaupt H. (2019). Engineering Genetic Predisposition in Human Neuroepithelial Stem Cells Recapitulates Medulloblastoma Tumorigenesis. *Cell Stem Cell* 25 433.e–446.e. 10.1016/j.stem.2019.05.013 31204176 PMC6731167

[B12] Johnson-Kerner B. Snijders Blok L. Suit L. Thomas J. Kleefstra T. Sherr E. H. (2020). ““*DDX3X*-Related Neurodevelopmental Disorder,”,” in *GeneReviews(§)*, eds Adam M. P. Ardinger H. H. Pagon R. A. Wallace S. E. Bean L. J. H. Mirzaa G. (Seattle (WA): University of Washington). 32852922

[B13] Kellaris G. Khan K. Baig S. M. Tsai I. C. Zamora F. M. Ruggieri P. (2018). A hypomorphic inherited pathogenic variant in *DDX3X* causes male intellectual disability with additional neurodevelopmental and neurodegenerative features. *Hum. Genom.* 12:11. 10.1186/s40246-018-0141-y 29490693 PMC5831694

[B14] Kumar P. Mohan V. Sinha R. A. Chagtoo M. Godbole M. M. (2015). Histone deacetylase inhibition reduces hypothyroidism-induced neurodevelopmental defects in rats. *J. Endocrinol.* 227 83–92. 10.1530/JOE-15-0168 26427529

[B15] Lai M. C. Lee Y. H. Tarn W. Y. (2008). The DEAD-box RNA helicase DDX3 associates with export messenger ribonucleoproteins as well as tip-associated protein and participates in translational control. *Mol. Biol. Cell* 19 3847–3858. 10.1091/mbc.e07-12-1264 18596238 PMC2526709

[B16] Lee C. S. Dias A. P. Jedrychowski M. Patel A. H. Hsu J. L. Reed R. (2008). Human DDX3 functions in translation and interacts with the translation initiation factor eIF3. *Nucleic Acids Res.* 36 4708–4718. 10.1093/nar/gkn454 18628297 PMC2504307

[B17] Lennox A. L. Hoye M. L. Jiang R. Johnson-Kerner B. L. Suit L. A. Venkataramanan S. (2020). Pathogenic *DDX3X* Mutations Impair RNA Metabolism and Neurogenesis during Fetal Cortical Development. *Neuron* 106 404.e–420.e. 10.1016/j.neuron.2020.01.042 32135084 PMC7331285

[B18] Linsalata A. E. He F. Malik A. M. Glineburg M. R. Green K. M. Natla S. (2019). *DDX3X* and specific initiation factors modulate FMR1 repeat-associated non-AUG-initiated translation. *EMBO Rep.* 20:e47498. 10.15252/embr.201847498 31347257 PMC6726903

[B19] Marvin A. R. Marvin D. J. Lipkin P. H. Law J. K. (2017). Analysis of Social Communication Questionnaire (SCQ) Screening for Children Less Than Age 4. *Curr. Dev. Dis. Rep.* 4 137–144. 10.1007/s40474-017-0122-1 29188169 PMC5684265

[B20] Maulik P. K. Mascarenhas M. N. Mathers C. D. Dua T. Saxena S. (2011). Prevalence of intellectual disability: a meta-analysis of population-based studies. *Res. Dev. Disabil.* 32 419–436. 10.1016/j.ridd.2010.12.018 21236634

[B21] Patmore D. M. Jassim A. Nathan E. Gilbertson R. J. Tahan D. Hoffmann N. (2020). *DDX3X* Suppresses the Susceptibility of Hindbrain Lineages to Medulloblastoma. *Dev. Cell* 54 455.e–470.e. 10.1016/j.devcel.2020.05.027 32553121 PMC7483908

[B22] Phung B. Cieśla M. Sanna A. Guzzi N. Beneventi G. Ngoc P. (2019). The X-Linked *DDX3X* RNA Helicase Dictates Translation Reprogramming and Metastasis in Melanoma. *Cell Rep.* 27 3573.e–3586.e. 10.1016/j.celrep.2019.05.069 31216476

[B23] Prezioso G. Giannini C. Chiarelli F. (2018). Effect of Thyroid Hormones on Neurons and Neurodevelopment. *Horm. Res. Paediatr.* 90 73–81. 10.1159/000492129 30157487

[B24] Ramaswami M. Taylor J. P. Parker R. (2013). Altered ribostasis: RNA-protein granules in degenerative disorders. *Cell* 154 727–736. 10.1016/j.cell.2013.07.038 23953108 PMC3811119

[B25] Richards S. Aziz N. Bale S. Bick D. Das S. Gastier-Foster J. (2015). Standards and guidelines for the interpretation of sequence variants: a joint consensus recommendation of the American College of Medical Genetics and Genomics and the Association for Molecular Pathology. *Genet. Med.* 17 405–424. 10.1038/gim.2015.30 25741868 PMC4544753

[B26] Snijders Blok L. Madsen E. Juusola J. Gilissen C. Baralle D. Reijnders M. R. (2015). Mutations in *DDX3X* Are a Common Cause of Unexplained Intellectual Disability with Gender-Specific Effects on Wnt Signaling. *Am. J. Hum. Genet.* 97 343–352. 10.1016/j.ajhg.2015.07.004 26235985 PMC4573244

[B27] Tuhan H. Abaci A. Cicek G. Anik A. Catli G. Demir K. (2016). Levothyroxine replacement in primary congenital hypothyroidism: the higher the initial dose the higher the rate of overtreatment. *J. Pediatr. Endocrinol. Metab.* 29 133–138. 10.1515/jpem-2015-0047 26244672

[B28] Wang X. Posey J. E. Rosenfeld J. A. Bacino C. A. Scaglia F. Immken L. (2018). Phenotypic expansion in *DDX3X* - a common cause of intellectual disability in females. *Ann. Clin. Transl. Neurol.* 5 1277–1285. 10.1002/acn3.622 30349862 PMC6186933

[B29] Yang Y. (2016). *Rating Scales For Children’s Developmental Behavior and Mental Health.* Beijing: People’s Medical Publishing House.

[B30] You J. Shamsi B. H. Hao M. C. Cao C. H. Yang W. Y. (2019). A study on the neurodevelopment outcomes of late preterm infants. *BMC Neurol.* 19:108. 10.1186/s12883-019-1336-0 PMC6542031 31146703

