# Supplementary material for: Expansion of Clinical and Genetic Spectrum of DDX3X Neurodevelopmental Disorder in 23 Chinese Patients
Source: Front Mol Neurosci. 2022 Mar 22;15:793001. doi: 10.3389/fnmol.2022.793001 (PMC8981727; doi:10.3389/fnmol.2022.793001)
Supplement: Supplementary file 1 [file Table_1.DOCX]

| Patient | Variant  origin | Variant  (NM_001193416) | *silico* analysis data for each missense variants | | | | | |  |
| --- | --- | --- | --- | --- | --- | --- | --- | --- | --- |
|  |  |  | GERP++ | CADD | SIFT | PROVEAN | PolyPhen-2 | M-CAP | GnomAD |
| Female 1 | *De novo* | c.1084C>G  p.(R362G) | 5.37 | 29.4 | Damaging | Damaging | Probably damaging | Damaging | - |
| Female 2 | *De novo* | c.635C>T;  p.(P212L) | 5.89 | 33 | Damaging | Damaging | Probably damaging | Damaging | - |
| Female 3 | *De novo* | c.1579delC  p.(H527fs*9) | / | / | / | / | / |  | - |
| Female 4 | *De novo* | c.1171-2A>C;  ? | / | / | / | / | / | / | - |
| Female 5 | *De novo* | c.369delA;  p.(N124Tfs*97) | / | / | / | / | / | / | - |
| Female 6 | *De novo* | c.1051C>G;  p.(R351G) | 3.67 | 26.5 | Damaging | Damaging | Probably damaging | Damaging | - |
| Female 7 | *De novo* | c.611C>T;  p.(T204I) | 5.89 | 31 | Damaging | Damaging | Probably damaging | Damaging | - |
| Female 8 | *De novo* | c.1595C>T;  p.(T532M) | 5.22 | 33 | Damaging | Damaging | Probably damaging | Damaging | - |
| Female 9 | *De novo* | c.749_756del  CTTTGAGG;  p.(A250Gfs*42) | / | / | / | / | / | / | - |
| Female 10 | *De novo* | c.136C>T;  p.(R46^*’^) | / | / | / | / | / | / | - |
| Female 11 | *De novo* | c.865-1G>A;  ? | / | / | / | / | / | / | - |
| Female 12 | *De novo* | c.693_695del  p.(Ala233del） | / | / | / | / | / | / | - |
| Female 13 | *De novo* | c.894C>A;  p.(C298^*^) | / | / | / | / | / | / | - |
| Female 14 | *De novo* | c.1633insT;  p.(F545Ffs*2) | / | / | / | / | / | / | - |
| Female 15 | *De novo* | c.1678_1680del;  p.(L560del) | / | / | / | / | / | / | / |
| Female 16 | *De novo* | c.1703C>T;  p.(P568L) | 5.28 | 34 | Damaging | Damaging | Probably damaging | Damaging | - |
| Female 17 | *De novo* | c.1679T>G;  p.(L560R) | 5.28 | 29.5 | Damaging | Damaging | Probably damaging | Damaging | - |
| Female 18 | *De novo* | c.1463G>A;  p.(R488H) | 5.22 | 29.8 | Damaging | Damaging | Probably damaging | Damaging | - |
| Female 19 | *De novo* | c.620_626  dupAAAGCA;  p.(His209Glnfs*88） | / | / | / | / | / | / | - |
| Female 20 | *De novo* | c.3G>A;  p.(M1I） | 4.34 | 24.7 | Damaging | Tolerable | Probably damaging | Damaging | - |
| Female 21 | *De novo* | c.1628C>G；  p.(S543^*^) | / | / | / | / | / | / | - |
| Female 22 | *De novo* | c.605G>C  p.(R202P) | 5.89 | 28.7 | Damaging | Damaging | Probably damaging | Damaging | - |
| Male 1 | Inherited from his mother | c.329G>A;  p.(R110H) | 5.75 | 23.8 | Damaging | Damaging | Probably damaging |  | - |

Table S1. In *silico* analysis of variants detected in *DDX3X*.GERP++, genomic evolutionary rate profling; CADD, combined annotation dependent depletion.
